# Brain Tumor Segmentation From Multi-Modal MR Images via Ensembling UNets

**DOI:** 10.3389/fradi.2021.704888

**Published:** 2021-10-21

**Authors:** Yue Zhang, Pinyuan Zhong, Dabin Jie, Jiewei Wu, Shanmei Zeng, Jianping Chu, Yilong Liu, Ed X. Wu, Xiaoying Tang

**Affiliations:** ^1^Department of Electrical and Electronic Engineering, Southern University of Science and Technology, Shenzhen, China; ^2^Laboratory of Biomedical Imaging and Signal Processing, The University of Hong Kong, Hong Kong, Hong Kong SAR, China; ^3^Department of Electrical and Electronic Engineering, The University of Hong Kong, Hong Kong, Hong Kong SAR, China; ^4^Tencent Music Entertainment, Shenzhen, China; ^5^School of Electronics and Information Technology, Sun Yat-Sen University, Guangzhou, China; ^6^Department of Radiology, The First Affiliated Hospital, Sun Yat-Sen University, Guangzhou, China

**Keywords:** segmentation, brain tumor, UNet, brain parcellation, ensemble

## Abstract

Glioma is a type of severe brain tumor, and its accurate segmentation is useful in surgery planning and progression evaluation. Based on different biological properties, the glioma can be divided into three partially-overlapping regions of interest, including whole tumor (WT), tumor core (TC), and enhancing tumor (ET). Recently, UNet has identified its effectiveness in automatically segmenting brain tumor from multi-modal magnetic resonance (MR) images. In this work, instead of network architecture, we focus on making use of prior knowledge (brain parcellation), training and testing strategy (joint 3D+2D), ensemble and post-processing to improve the brain tumor segmentation performance. We explore the accuracy of three UNets with different inputs, and then ensemble the corresponding three outputs, followed by post-processing to achieve the final segmentation. Similar to most existing works, the first UNet uses 3D patches of multi-modal MR images as the input. The second UNet uses brain parcellation as an additional input. And the third UNet is inputted by 2D slices of multi-modal MR images, brain parcellation, and probability maps of WT, TC, and ET obtained from the second UNet. Then, we sequentially unify the WT segmentation from the third UNet and the fused TC and ET segmentation from the first and the second UNets as the complete tumor segmentation. Finally, we adopt a post-processing strategy by labeling small ET as non-enhancing tumor to correct some false-positive ET segmentation. On one publicly-available challenge validation dataset (BraTS2018), the proposed segmentation pipeline yielded average Dice scores of 91.03/86.44/80.58% and average 95% Hausdorff distances of 3.76/6.73/2.51 mm for WT/TC/ET, exhibiting superior segmentation performance over other state-of-the-art methods. We then evaluated the proposed method on the BraTS2020 training data through five-fold cross validation, with similar performance having also been observed. The proposed method was finally evaluated on 10 in-house data, the effectiveness of which has been established qualitatively by professional radiologists.

## 1. Introduction

**Figure 1:**
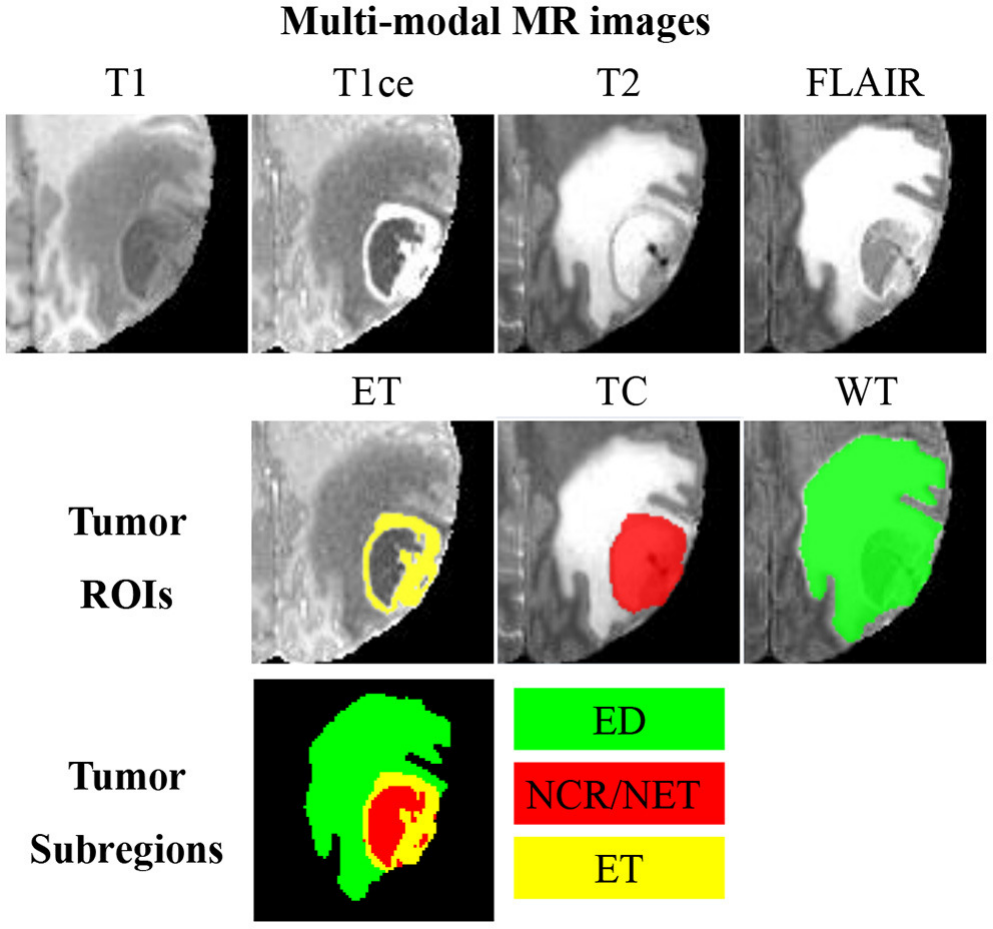
An example of multi-modal MR images, three partially-overlapping tumor ROIs (ET, TC, and WT), as well as three non-overlapping tumor subregions (ED, NCR/NET, and ET). MR images of different modalities can best display different ROIs; visualizations of ET, TC, and WC are, respectively, the best in T1ce, T2, and FLAIR.

Glioma is one of the lethal brain malignancies, and it may severely damage the nervous system and endanger patients (1). Early diagnoses, treatments and interventions of glioma are hot research topics due to the high incidence of glioma (2). Surgery is the primary treatment method for glioma, and clear boundary detection is the prerequisite for a successful glioma surgery. Besides, tumor volume analysis is also very essential for evaluating the progression of the disease. In clinical studies, it is challenging to segment glioma from a single-modal magnetic resonance (MR) image as the image intensity may be obscured by partial volume effects or bias field artifacts (3–6). As such, glioma is typically detected using multi-modal MR sequences, including T1-weighted (T1), contrast-enhanced T1-weighted (T1ce), T2-weighted (T2), and fluid attenuation inversion recovery (FLAIR). An example of multi-modal MR sequences and the corresponding manual segmentation of glioma is illustrated in Figure 1. Glioma consists of three non-overlapping subregions: edema (ED), enhancing tumor (ET), and necrotic core and non-enhancing tumor (NCR/NET). Different subregions reflect different biological properties. From the aforementioned three subregions, another three regions of interest (ROIs) that are more commonly used in literature can be formed, namely whole tumor (WT), tumor core (TC), and ET. WT denotes the union of all three subregions. TC covers both NCR/NET and ET.

Quantitative analyses of the aforementioned three ROIs provide critical information for disease diagnosis, surgical planning, and prognosis (3), for which accurate segmentation of brain tumor and the corresponding ROIs are essential. Labels manually traced by professional radiologists are regarded as the gold standard. However, manual tracing is excessively impractical in most clinical workflows because it is labor-intensive and subjective (7). Fully-automated brain tumor segmentation approaches are thus urgently needed.

In one of our previous works, Wu et al. divided a brain into five parcellations: gray matter, white matter, cerebrospinal fluid, lateral ventricles, and skull (8). We conjecture such prior location information is beneficial for brain tumor segmentation, especially in deep learning frameworks which are purely data-driven (9, 10). Deep learning has become the mainstream for brain tumor segmentation in recent years (11). Training and testing utilizing 3D patches or 2D slices are adopted in most deep learning architectures a representative one of which is UNet (12, 13). Typically, 3D patch-based UNets perform better than 2D slice-based ones since there is additional context information in the direction orthogonal to 2D slices (14). It is nevertheless challenging to identify the most suitable patch sizes for 3D UNets to ensure sufficient learning of all features, especially the boundary features. More specifically, 3D UNets with small patch sizes may be unable to capture sufficient features, whereas GPU may be unable to support larger patch sizes.

To resolve the aforementioned issues, we employ a multi-input UNet (MI-UNet) that jointly uses MR images and brain parcellation (BP) as the input. We also make use of a joint 3D+2D training strategy in the brain tumor segmentation task and adopt ensemble to improve the segmentation accuracy; three UNets with different inputs are ensembled. In summary, the contributions of this work are four-fold: (1) We demonstrate that BP is useful for UNet-based brain tumor segmentation. Together with joint 3D+2D and ensemble strategies, the segmentation accuracy further gets enhanced. (2) Our method achieves superior segmentation performance over state-of-the-art methods on a publicly-available dataset. (3) Our method achieves visually promising results on 10 in-house data. (4) We release our pre-trained models and describe in great detail how to reproduce all results presented in this work at Dockerhub and Github.

The remainder of this paper is organized as follows. We describe related works in section 2. The detailed procedure of the proposed method is presented in section 3. The datasets and evaluation criteria are presented in section 4. We then report evaluations and experimental results in section 5. Finally, section 6 concludes the paper.

## 2. Related Work

Recently, deep learning has made remarkable advances in various medical image segmentation tasks, especially when there exist large-scale training data. The multi-modal Brain Tumor Segmentation (BraTS) challenge has released a large amount of pre-operative multi-modal MR images and the corresponding manual annotations of brain tumor (3–6). Benefited from this large dataset, convolutional neural network (CNN) has quickly dominated the fully-automated brain tumor segmentation field (15–29).

CNN methods can be either 2D slice-based or 3D patch-based. In 2D CNN methods, a 3D volume is divided into multiple 2D slices and brain tumor is independently predicted for each slice (15–21). For example, Caver et al. employed three 2D UNets to separately segment WT, TC, and ET in a slice-by-slice manner (18). Choudhury et al. proposed to separately train three multi-class segmentation models, respectively, on axial, coronal, and sagittal slices and use majority voting to make the final predictions (19). McKinley et al. presented a novel DeepSCAN architecture by embedding a pooling-free DenseNet into a UNet. They also applied their model, respectively, on three planes and then used majority voting to make the final predictions. This multi-view fusion technology of 2D CNN results has also been widely used in other tasks (30–32), named 2.5D CNN. Compared with 2D slice-based method, 3D patch-based methods are relatively more widely used in the brain tumor segmentation task as they can capture characteristics of the volume data in the extra dimension (22–29). Feng et al. demonstrated that 3D UNet performed slightly better than DenseNet in terms of TC segmentation (24). Luo et al. presented a hierarchical decoupled CNN (HDC-Net) by decoupling the convolution in the spatial and channel dimensions (25). Isensee et al. suggested that a well-trained UNet is indeed very challenging to outperform, and their released code on UNet performed much better than existing state-of-the-art methods on many medical segmentation tasks (33).

Moreover, there are some studies employing other types of deep learning networks for brain tumor segmentation, including generative adversarial network (GAN), transformer, and capsule neural network. For instance, Nema et al. designed a network architecture, named residual cyclic unpaired GAN (RescueNet), based on residual and mirroring principles (34). Wang et al. exploited transformer in 3D CNN for brain tumor segmentation and proposed a novel network named TransBTS based on an encoder-decoder structure (35). Aziz et al. optimized a network based on the capsule neural network, named SegCaps, to achieve accurate glioma segmentation from MR images (36).

In addition to the aforementioned 2D slice-based and 3D-patch based CNN methods, efforts have also been made in exploring additional input information so as to enhance the segmentation performance. Prior knowledge in the form of template or shape has been successfully used for brain segmentation in some previous studies. Dalca et al. proposed an inference algorithm by modeling intensity, shape, and spatial distribution of pathologies to capture their anatomical prior, for an automatic segmentation of cerebrovascular pathologies in brain MR images (37). Wu et al. proposed and validated a multi-atlas and diffeomorphism guided 3D fully convolutional network for brain segmentation (38). Moreover, there have been studies employing shape prior. Mahbod et al. extracted level set based context feature as an additional input of a neural network for automatic brain segmentation (39). Brusini et al. proposed a deep learning based hippocampus segmentation framework embedding statistical shape of the hippocampus as context information (40). In addition to template and shape prior, some studies incorporated BP in their segmentation pipelines. Kao et al. employed a pre-defined brain atlas to obtain BP and demonstrated that using BP as an additional input to CNN can improve the brain tumor segmentation accuracy (23). We previously also demonstrated that an additional BP input can improve the stroke lesion segmentation accuracy (41). Unlike most deep learning-based segmentation methods utilizing only MR images as the input, we proposed MI-UNet with BP as an additional input. MI-UNet performed significantly better than UNet with MR images being the single input, in both 2D and 3D settings. Our proposed MI-UNet even outperformed some existing state-of-the-art methods (41).

Recently, some researchers have tried to combine 2D and 3D CNNs in a unified framework. For example, Li et al. proposed a hybrid densely-connected UNet (H-DenseUNet) that used a 2D DenseUNet to capture intra-slice features and then a 3D DenseUNet to capture inter-slice features (42). Jia et al. proposed a Hybrid Discriminative Network (HD-Net), jointly making use of a 3D segmentation decoder and a 2D boundary decoder (43). One of our previous works presented a joint 3D+2D strategy by using the probability map from a pre-trained 3D UNet as an additional input to a subsequent 2D UNet (44). We then extended this joint 3D+2D strategy to the pancreas segmentation task and successfully demonstrated that this strategy can reduce both false-negative and false-positive predictions (14).

Compared to a single CNN model, ensemble provides a more robust solution with less variance (45). The ensemble strategy has also been employed in the brain tumor segmentation methods. For example, Sun et al. independently trained three models (CA-CNN, DKFZ Net, and 3D UNet) and then used majority voting to obtain the final brain tumor segmentation (22). Kao et al. trained eight models with different network architectures, input channels, as well as convolutional kernels (23). They then obtained the final brain tumor segmentation based on an average of the outputted probability maps from all eight models.

## 3. Method

**Figure 2:**
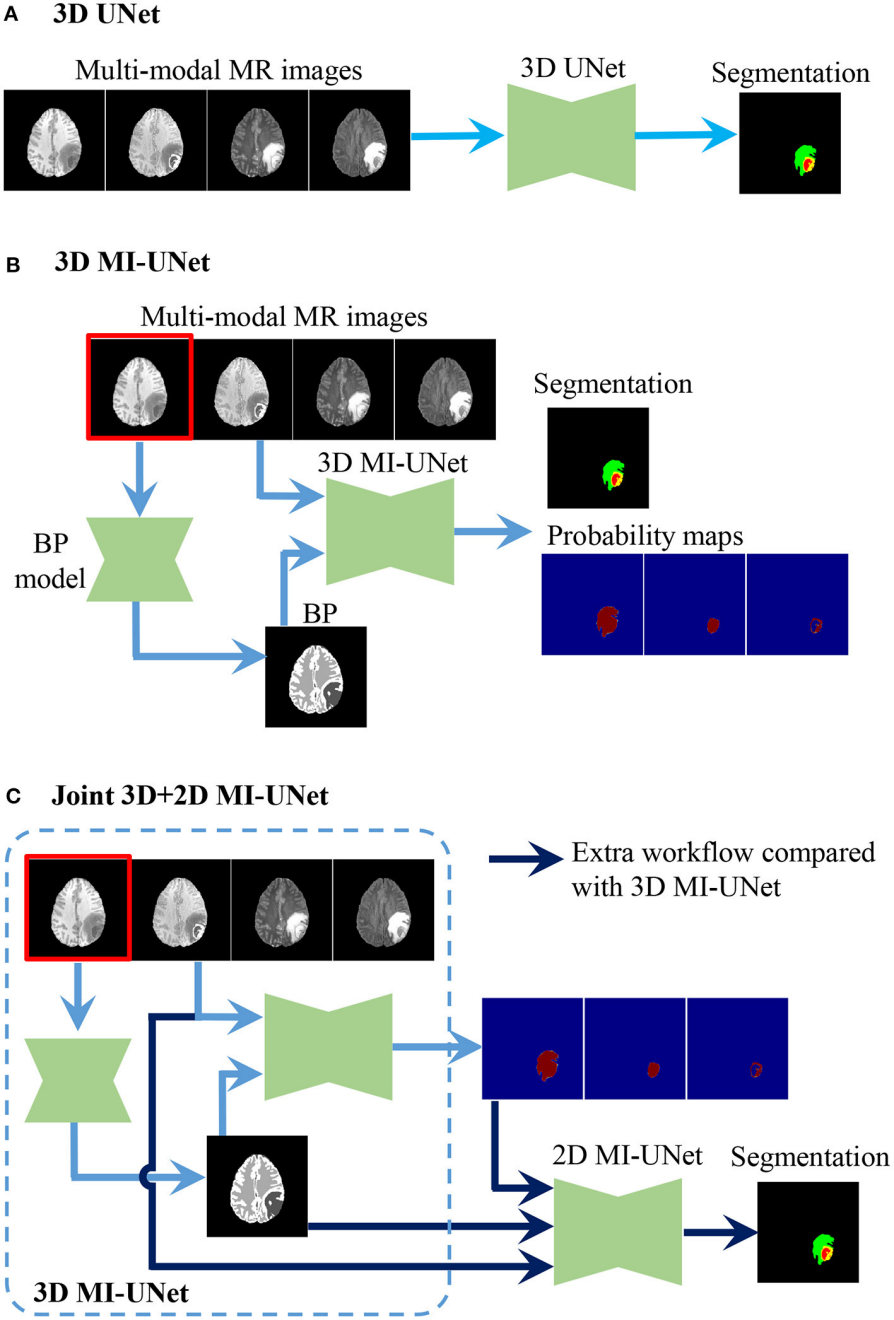
Procedure of the three UNets employed in the proposed method. **(A)** 3D UNet. **(B)** 3D MI-UNet. **(C)** Joint 3D+2D MI-UNet.

UNet is a widely used method for biomedical image segmentation, which has an encoder-decoder architecture with skip connections (12, 13). We utilize UNet as our baseline and then explore the effectiveness of MI-UNet, joint 3D+2D, as well as ensemble. There are three UNets separately trained in the proposed method. As shown in Figure 2, the major difference among these three UNets lies in the number of the input channels. To be specific, model 1, model 2, and model 3, respectively, have four, five, and eight input channels.

### 3.1. 3D UNet

Considering the fact that different tumor subregions are better visible in MR images of different modalities (46), four different-modal MR images are jointly used in our proposed pipeline, including T1, T1ce, T2, and FLAIR. As shown in Figure 1, the provided labels for training are the three non-overlapping tumor subregions, namely ED, NCR/NET, and ET. However, evaluation of the segmentation is based on the three partially-overlapping tumor ROIs, namely WT, TC, and ET. We use the overlapping ROIs rather than the non-overlapping subregions as the expected output because it has been previously suggested that this strategy can obtain better segmentation performance (47, 48). The hyper-parameters of our 3D UNet are fully automatically generated by nnUNet (33).

### 3.2. 3D MI-UNet

**Figure 3:**
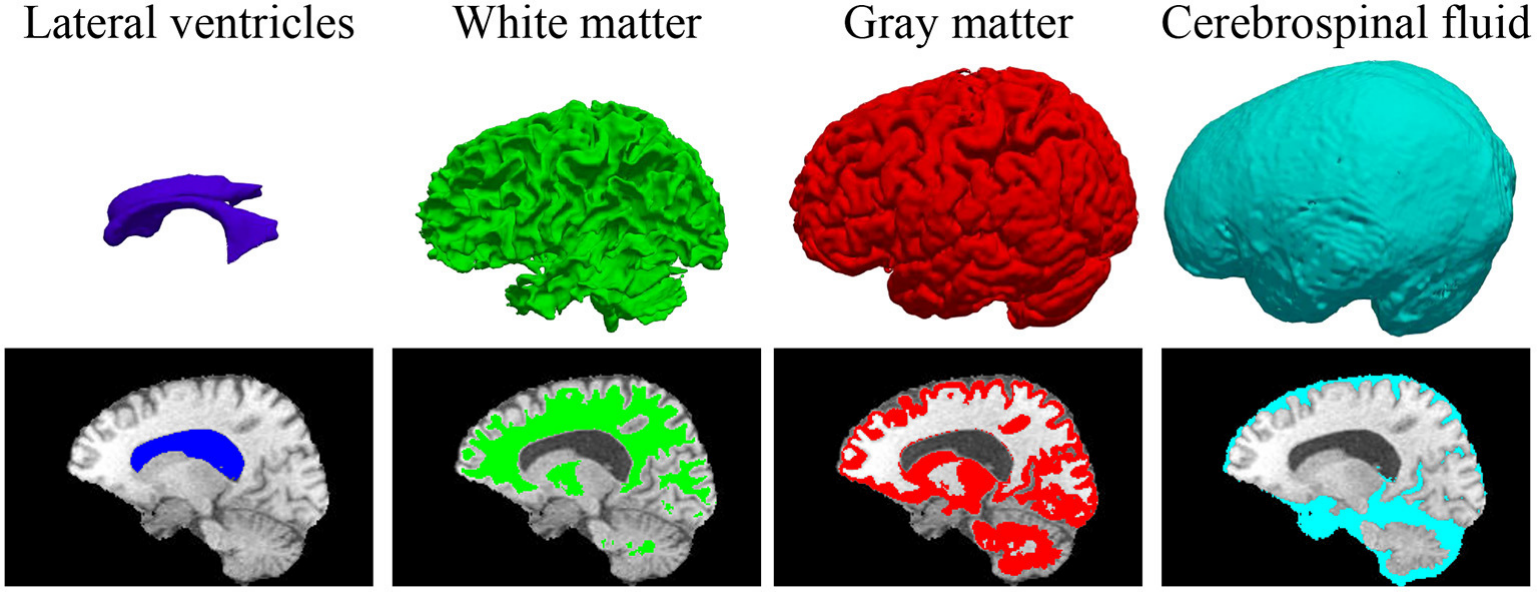
3D displays and overlayed T1 MR images of the four brain parcellations for a representative case from the BraTS dataset.

In some previous works (23, 41), BP has been demonstrated to be useful in improving the brain lesion segmentation accuracy. We train a 3D MI-UNet that uses not only the multi-modal MR images but also the corresponding BP as the inputs. A BP model is trained using a dataset published in one of our previous works (8), which outputs a five-class BP with a T1 MR image as the input. All MR images of the BraTS dataset have already been skull-stripped. As such, the BP of T1 MR images from the BraTS dataset consists of only four subregions: lateral ventricles, white matter, gray matter, and cerebrospinal fluid, an example of which is shown in Figure 3. We use this model to obtain BPs for all training and validation data.

### 3.3. Joint 3D+2D MI-UNet

**Figure 4:**
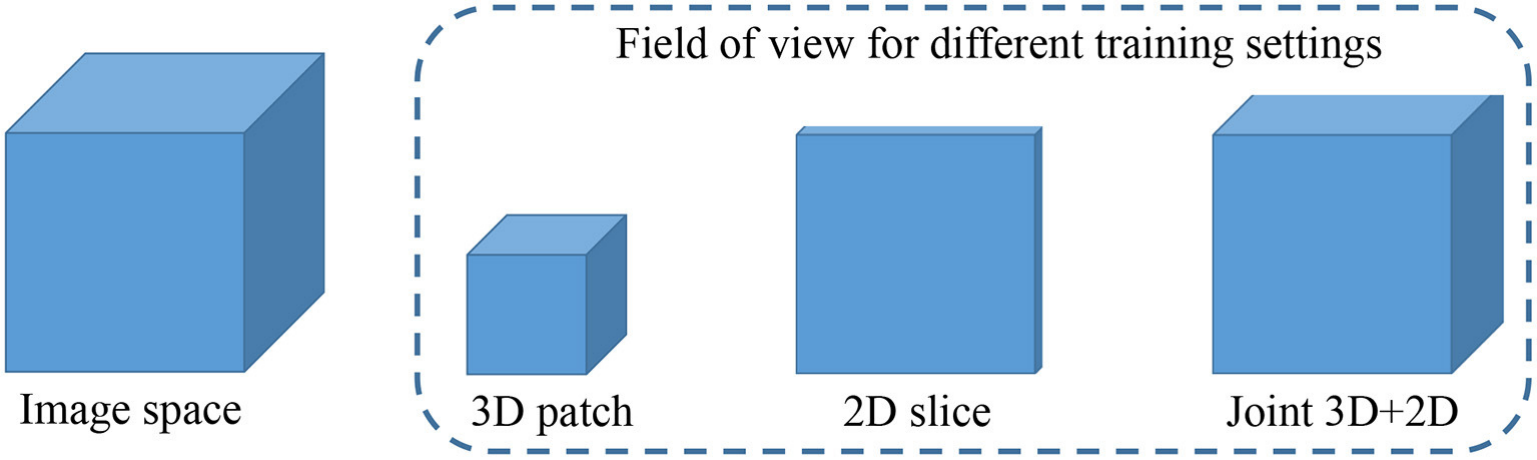
Illustration of image space and the filed of view for different training settings. Both 3D patch-based and 2D slice-based methods has relative smaller field of views than the proposed joint 3D+2D method.

In this brain tumor segmentation task, the aforementioned 3D MI-UNet also outputs four probability maps that, respectively, represent the probability that each voxel belongs to the background, WT, TC, and ET. We firstly use the trained 3D MI-UNet to predict the training data and then use the 2D slices of the four multi-modal MR images, BP, and probability maps of WT, TC, and ET as the inputs to train a subsequent 2D MI-UNet. This strategy is named joint 3D+2D MI-UNet, the effectiveness of which has already been identified in some other segmentation tasks (8, 14, 44). As shown in Figure 4, this joint 3D+2D MI-UNet has a larger field of view (FOV) than either 3D patch-based UNet or 2D slice-based UNet.

### 3.4. Model Ensemble and Post-processing

In the testing stage, we obtain three segmentation results independently from the three aforementioned UNet models (Figure 2). To reduce model variance and further improve the segmentation performance, we use a weighted voting strategy to obtain a fused segmentation.

One of the most challenging parts in the brain tumor segmentation task is to distinguish small vessels (that should be labeled as either ED or NCR) from ET (26). To alleviate such false-positive ET segmentation, we post-process the fused segmentation by replacing ET with NCR/NET when the volume of the predicted ET is less than a specific threshold (500 voxels), which was originally proposed by Ding's group (47) and has been adopted in many previous works (48–50).

### 3.5. Implementation Details

We set the epoch number to be 1,000 in all three UNet training. We release an end-to-end implementation of this work at Dockerhub^1^, in terms of both pre-trained models and codes. Please note our in-house multi-modal MR data have varied image sizes and orientations. Pre-processing for our in-house data is the same as that for the BraTS dataset (3). Firstly, we conduct a coregistration to align multi-modals image with different sizes. After the coregistration, the sizes of T1, T2, FLAIR are the same as that of T1ce. Next, we normalize all images to be of a spatial resolution 1 × 1 × 1 mm^3^ and apply skull-stripping using the FMRIB Software Library (51–53). The codes and an example have been presented at Github^2^.

## 4. Dataset and Evaluation Metrics

### 4.1. Dataset

Details of the three datasets used in this work are tabulated in Table 1. The first two datasets are provided by the BraTS2018 and BraTS2020 challenge organizers (3–6). Please note the ground truth labels of the BraTS2018 and BraTS2020 validation data are unavailable, and the predicted segmentations are uploaded to the CBICA's Imaging Processing Portal (IPP) for evaluation^3^. In addition to those BraTS datasets, we also collect multi-modal MR images from 10 patients at The First Affiliated Hospital of Sun Yat-Sen University, to further qualitatively evaluate the proposed segmentation framework. We will release these clinical MR data upon this manuscript getting accepted.

**Table 1:** The three datasets used in this work.

**Dataset**	**Training**	**Validation**	**Experiment setting**
BraTS2018	285	66	285 for training, 66 for validation
BraTS2020	369	125	Five-fold on the 369 training dataset
SYSU	0	10	Validation only

### 4.2. Evaluation Metrics

The Dice score (DSC) is the most widely used evaluation metric to quantify the performance of medical image segmentation. It is defined as


(1) $$\begin{array}{l} \text{DSC}\left(\text{G},\text{ R}\right)=\frac{2|\text{G}\cap \text{R}|}{\left|\text{G}|+|\text{R}\right|}, \end{array}$$

where **R** denotes an automated segmentation result, **G** denotes the corresponding ground truth segmentation, and **G**∩**R** denotes the overlap between **G** and **R**. The operator | · | returns the number of pixels (or voxels in 3D) contained in a region which is proportional to the physical volume of the considered region.

Let *S*(**A**) denote the set of surface vertices of a 3D volume **A**, the shortest distance of any vertex *v* to *S*(**A**) is defines as


(2) $$\begin{array}{l} d\left(v,S\left(\text{A}\right)\right)=\underset{s_{\text{A}}\in S\left(\text{A}\right)}{\min}\|v-s_{\text{A}}\|, \end{array}$$

where || · || denotes the Euclidean distance, with a greater value indicating a higher error.

Hausdorff distance (HD) measures how far the surface vertices of two binary masks lie from each other. It is defined as


(3) $$\begin{array}{l} \text{HD}\left(\text{G},\text{ R}\right)=\text{max }\{\underset{s_{\text{G}}}{\sup}d\left(s_{\text{G}},S\left(\text{R}\right)\right),\underset{s_{\text{R}}}{\sup}d\left(s_{\text{R}},S\left(\text{G}\right)\right)\}, \end{array}$$

where *s*_**R**_ and *s*_**G**_, respectively, denote the surface vertices of an automated segmentation result **R** and the corresponding ground truth segmentation **G**, and sup denotes the supremum. To avoid potential issues induced by small noisy segmentations, HD is modified to be a robust version by using its 95th percentile, namely H95, instead of the maximum distance.

The DSC and H95 of WT, TC, and ET are calculated to evaluate the performance of our brain tumor segmentation task, being consistent with previous studies (3–6).

## 5. Results

### 5.1. Comparison With Other Methods on the BraTS2018 Validation Dataset

Table 2 compares the proposed method with some published ones. All these methods were trained using the BraTS2018 training dataset and tested on the BraTS2018 validation dataset. The compared methods can be roughly categorized into 2D slice-based ones (17–20) and 3D patch-based ones (22–26). Typically, 3D patch-based methods perform better than 2D slice-based ones. However, some 2D slice-based methods with a multi-view ensemble strategy (19, 20) achieve even better segmentation results than 3D patch-based methods (22, 23), indicating the potential of 2.5D slice-based methods as they can capture the entire FOV in each single plane (see Figure 4). All methods show better performance in WT segmentation than both TC and ET segmentation in terms of DSC. This is because a large-object segmentation task can easily obtain a higher DSC than a small-object segmentation task. It can be nevertheless observed that H95 will not be affected by such volume bias. Feng et al. achieves the best H95 of TC (5.34 mm) using six UNets (24), but their H95 of ET is relatively worse than other methods. Luo et al. achieves the best Dice/H95 of ET (81.5%/2.42 mm) using the singe HDC-Net (25), but their Dice/H95 of TC (84.3%/8.76 mm) are relatively worse than other methods.

**Table 2:** Comparison of different brain tumor segmentation results obtained on the BraTS2018 validation dataset.

	**Dice score (DSC)[%]**					**95% Hausdorff distance (H95) [mm]** **↓**			
	**No. of models**	**WT**	**TC**	**ET**	**Overall**	**WT**	**TC**	**ET**	**Overall**
M-UNet (17)	1	87.00	72.00	66.00	75.00	6.73	15.74	7.56	10.01
CA+DFKZ+UNet (22)	3	90.44	80.52	74.94	81.97	6.33	6.37	2.78	5.16
2D UNet (18)	3	89.1	80.9	79.3	83.1	6.99	8.96	4.12	6.69
DeepMedic+UNet (23)	26	90.5	81.3	78.8	83.53	4.32	7.56	3.81	5.17
DeeplabV3+ (19)	3	90.48	84.14	76.49	83.70	5.19	7.24	3.78	5.40
3D UNet (24)	6	90.59	83.36	78.73	84.23	4.02	**5.34**	3.96	4.44
DeepSCAN (20)	6	90.28	85.40	79.47	84.98	—	—	—	—
HDC-Net (25)	1	89.0	84.3	**81.5**	84.93	4.59	8.76	**2.42**	5.26
No new-Net (26)	10	90.83	85.44	81.05	85.76	4.97	7.04	2.51	4.85
The proposed	4	**91.03**	**86.44**	80.58	**86.02**	**3.76**	6.73	2.51	**4.33**

*The results are reported as mean and are directly copied from the original papers. Bold numbers highlight the best performance in each column*.

↓ *indicates that a smaller value represents a better performance*.

Although a single 3D model such as HDC-Net can deliver promising results, most existing methods employ ensemble strategies to further improve the accuracy. With an ensemble of three UNets, the proposed method exhibits outstanding segmentation performance in terms of both DSC and H95 (86.02% and 4.34 mm), performing even better than some methods that ensemble more models (23, 24, 26). Furthermore, the proposed method obtains the highest DSC and lowest H95 in WT segmentation. Our UNet is implemented based on codes released by no-new-Net (26), but our method achieves a competitive segmentation accuracy with fewer models, which clearly demonstrates the effectiveness of the three strategies that we use: (1) MI-UNet, (2) joint 3D+2D, (3) ensemble and post-processing.

### 5.2. Ablation Analysis on the BraTS2018 Validation Dataset

Table 3 evaluates the importance of each component in the proposed method. We would like to kindly point out that CNN architecture design may involve fine-tuning a vast set of parameters. Considering most existing methods are based on UNet (17, 18, 22–24, 26), we simply use UNet as our baseline to emphasize our core contributions and findings: (1) BP can improve both DSC and H95 of WT segmentation. (2) The joint 3D+2D strategy can further improve the H95 of WT segmentation. (3) An ensemble of all the three UNets can obtain the best performance in all metrics (DSC/H95 of WT/TC/ET).

**Table 3:** Ablation analysis results of the proposed method on the BraTS2018 validation data.

	**Dice score (DSC)**				**95% Hausdorff distance (H95)** **↓**			
	**WT (%)**	**TC (%)**	**ET (%)**	**Overall (%)**	**WT (mm)**	**TC (mm)**	**ET (mm)**	**Overall (mm)**
3D UNet	90.79	86.12	78.95	85.28	4.40	7.12	2.93	4.82
3D MI-UNet	91.02	85.86	78.97	85.28	4.12	6.82	3.18	4.70
Joint 3D+2D MI-UNet	90.92	85.49	76.53	84.30	**3.76**	7.32	3.03	4.71
Ensemble	**91.03**	**86.44**	78.95	85.47	**3.76**	**6.73**	2.93	4.47
Ensemble+post-processing	**91.03**	**86.44**	**80.58**	**86.02**	**3.76**	**6.73**	**2.51**	**4.33**

*Bold numbers are the best values*.

We obtain an average DSC of 90.79/86.12/78.95% and an average H95 of 4.40/7.12/2.93 mm in WT/TC/ET segmentation using our baseline UNet (33). Apparently, UNet is indeed a strong baseline, the overall performance of which (85.28%/4.82 mm) is even better than the best single model in Table 2 [HDC-Net: 84.93%/5.26 mm (25)] in terms of DSC/H95. As shown in Figure 2, the only difference between 3D UNet and 3D MI-UNet is the additional input channel of BP. It can be observed from Table 3 that MI-UNet can improve the WT segmentation accuracy from 90.79%/4.40 mm to 91.02%/4.12 mm in terms of DSC/H95. The joint 3D+2D MI-UNet can significantly reduce the H95 of WT from 4.12 mm to 3.76 mm (p-value < 0.05). However, this joint strategy may impair the segmentation performance of TC/ET. As such, we set the ensemble weights of 3D UNet, 3D MI-UNet, and joint 3D+2D MI-UNet to be 0/0.5/0.5, 0/0.5/0.5/, 1/0/0 for WT/TC/ET, and sequentially assign the final segmentation of WT, TC, and ET in the ensemble step. The final segmentation after post-processing performs better than each individual model in respect of both DSC and H95 for WT/TC/ET segmentation.

### 5.3. Five-Fold Cross Validation on the BraTS2020 Training Dataset

**Figure 5:**
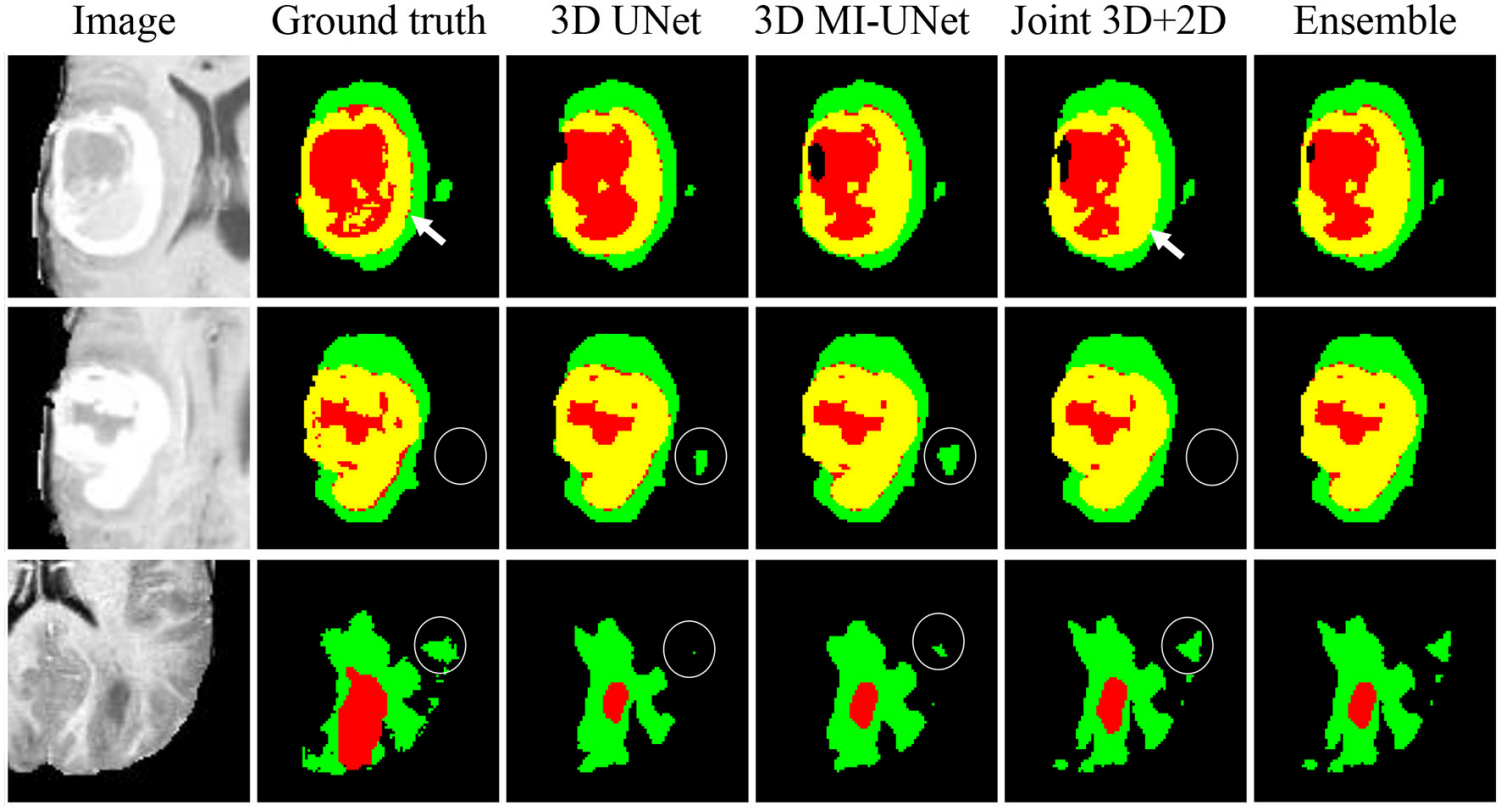
Three representative examples of segmentation results from five-fold cross validation on the BraTS2020 dataset.

We further conduct five-fold cross validation experiments on the training dataset of BraTS2020, which contains 369 multi-modal MR images and the corresponding ground truth tumor segmentation. As shown Figure 5, the joint 3D+2D MI-UNet can reduce some false-positive and false-negative WT segmentation (see white circles in the last two rows). However, it mis-assigns some disconnected ET as TC (see white arrows in the first row). Thus, we adopt the weighted ensemble strategy that successively assigns WT from the joint 3D+2D MI-UNet and the fusion of TC and ET, respectively, from the 3D UNet and 3D MI-UNet as the final segmentation.

Table 4 analyzes the impact of each component in the proposed method for the BraTS2020 dataset. The proposed method achieves an average DSC of 91.72/87.55/81.75% for WT/TC/ET and an average H95 of 4.67/4.55 mm for WT/TC on the BraTS2020 training dataset, which are similar to those obtained on the BraTS2018 validation dataset (average DSC: 91.03/86.44/80.58% for WT/TC/ET and average H95: 3.76/6.73 mm for WT/TC). However, there is a huge difference in H95 of the ET segmentation (2.51 vs. 22.94 mm, listed on Tables 3, 4). This is because that BraTS2020 has changed their evaluation criteria for empty labels. Given an empty ET in the ground truth and a false-positive segmentation, the BraTS2018 criteria will skip this case when calculating the average value. However, the BraTS2020 criteria will assign the H95 value for such case as 373.13 mm. There are 27 cases with empty ET in the ground truth segmentation among the 369 BraTS2020 training data, but there is no empty WT/TC. To make a fair comparison, all evaluation results are obtained through CBICA's IPP.

**Table 4:** Ablation analysis results of the proposed method on the BraTS2020 training data via five-fold cross validation.

	**Dice score (DSC)**				**95% Hausdorff distance (H95)** **↓**			
	**WT (%)**	**TC (%)**	**ET (%)**	**Overall (%)**	**WT (mm)**	**TC (mm)**	**ET (mm)**	**Overall (mm)**
3D UNet	91.48	87.35	78.87	85.90	6.39	7.18	26.40	13.32
3D MI-UNet	91.66	87.37	79.21	86.08	5.64	5.63	**21.90**	11.06
Joint 3D+2D MI-UNet	91.38	87.10	77.31	85.24	4.70	4.64	27.91	12.42
Ensemble	**91.72**	**87.55**	79.20	86.16	**4.67**	**4.55**	**21.90**	**10.37**
Ensemble+post-processing	**91.72**	**87.55**	**81.75**	**87.01**	**4.67**	**4.55**	22.94	10.72

*Bold numbers are the best values*.

**Figure 6:**
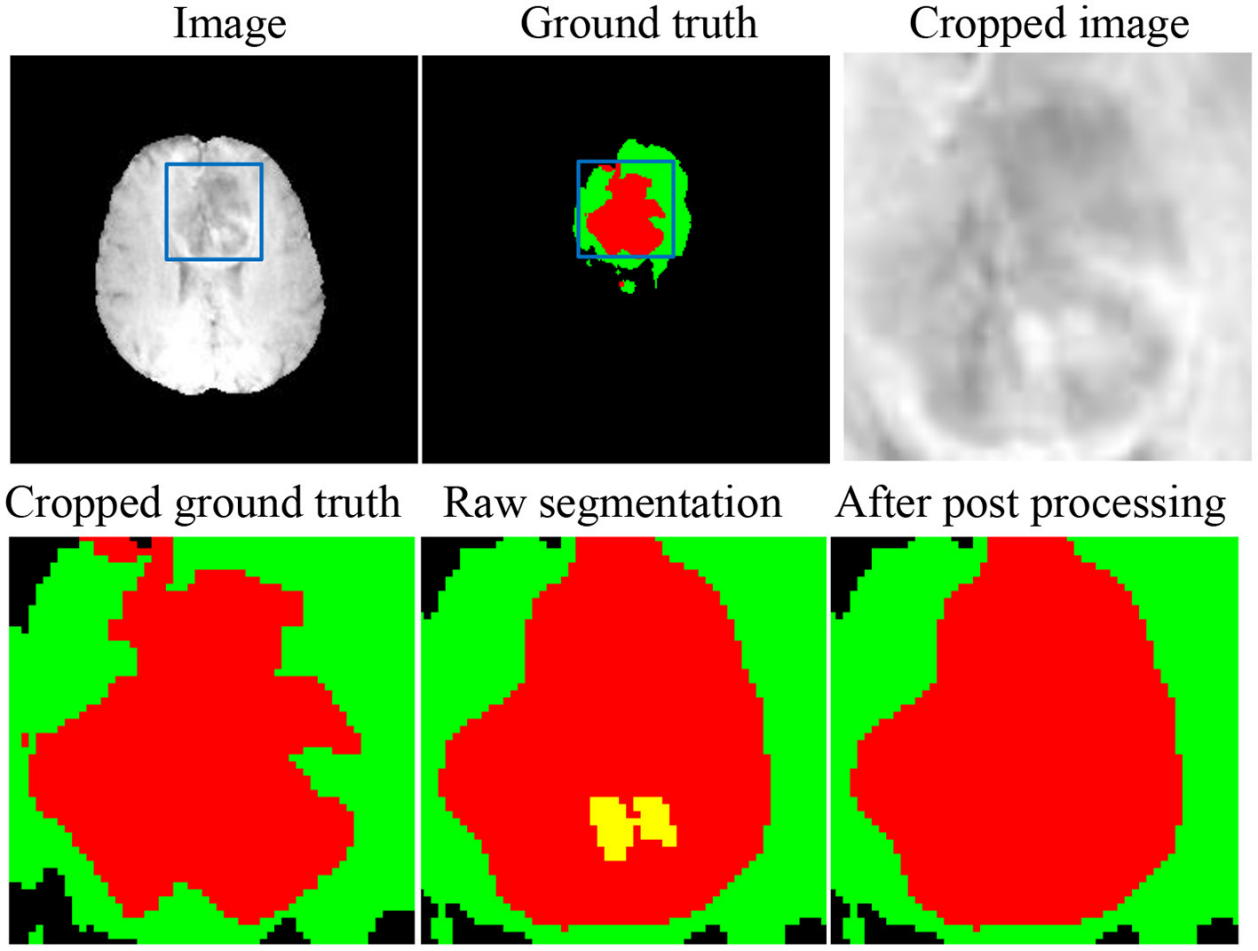
An example to demonstrate the influence of the post-processing.

As shown in Figure 6, there are still small false-positive ET segmentation when the ground truth is empty. The post-processing strategy described in section 3.4 can improve the DSC of ET segmentation from 0 to 1 for these cases.

### 5.4. Validation Results on In-House Data

**Figure 7:**
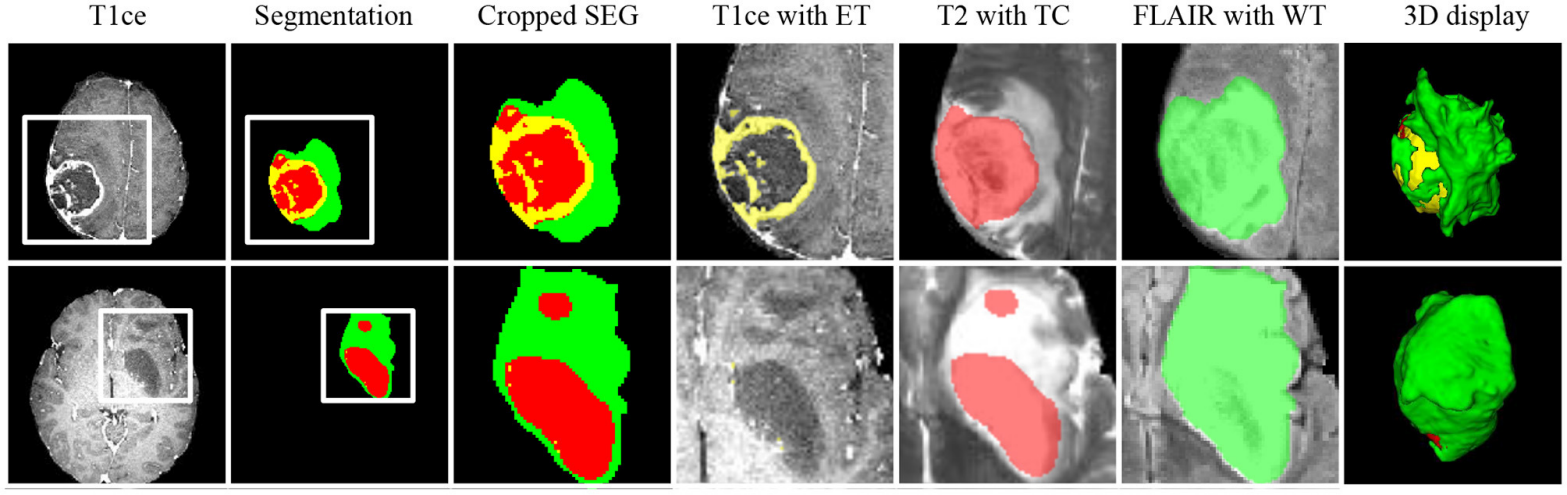
Representative cases from our in-house dataset of the two grades of tumors (high grade and low grade). From left to right: T1ce, automated tumor segmentation, zoomed-in segmentation, T1ce overlaid with ET, T2 overlaid with TC, FLAIR overlaid with WT, 3d display of tumor segmentation. ET, enhancing tumor; TC, tumor core; WT, whole tumor.

Figure 7 shows two representative slices of the final segmentation results from two grades of tumors (high grade and low grade). Two professional radiologists visually check the segmentation accuracy of the 10 in-house data and rate the potential clinical value. All segmentation results on the 10 data are considered as being satisfactory in terms of clinical applicability. The edema range of the tumor determines the margin of surgical resection (54, 55), since it is desirable to remove tumor as much as possible to avoid recurrence. ET is highly related to treatment planning, and the prognosis of patients with large edema (WT-TC) volumes may be relatively poor.

## 6. Discussion and Conclusion

Brain tumor segmentation plays a vital role in disease diagnosis and surgery planning. In this paper, we present an ensemble model that takes advantages of three individual models (3D UNet, 3D MI-UNet, and joint 3D+2D MI-UNet). UNet has been identified to perform very well on medical image segmentation (12, 26, 42), and changes in the network architecture may not bring much improvement. Therefore, we aim to address the tumor segmentation problem from aspects other than network architecture design. Our goal is to provide a robust, well-performing, and relatively simple and straightforward tumor segmentation pipeline, which may be more likely to adopted in routine clinics.

We demonstrate that MI-UNet and joint 3D+2D MI-UNet are highly beneficial for improving the WT segmentation accuracy and the ensemble results can achieve superior segmentation performance on the BraTS2018 dataset over some state-of-the-art methods. We further evaluate the proposed method on the BraTS2020 dataset and a small-scale in-house dataset.

In terms of computation time, the proposed method takes roughly 80 s to provide the final brain tumor segmentation for a multi-modal MR image set of size 240 × 240 × 155. This is relatively not a real-time task. As such, it is meaningful to obtain more accurate segmentation at the cost of a relatively longer running time.

We have released the pre-trained models, codes, and detailed description documents at Github and Docker, which can help researchers easily reproduce all results reported in this work. In the future, we will design a more user-friendly toolbox with graphic interface. That will further improve the utility and applicability of our proposed pipeline, especially for clinicians.

There are several potential limitations of this work. Firstly, we only evaluate the proposed method on 10 real-life clinical cases, and there is no manual annotation for that dataset. Thus we cannot conduct quantitative evaluations on those data but only qualitative assessments. In the future, we will collect more real-life data and provide manually-traced ground truth segmentation labels. That may better evaluate the clinical impact of our proposed method. Secondly, we only employ our proposed training strategy on UNet. To establish a generalized perspective, one of our future research plans is to further validate our proposed strategy on other deep learning models such as Deeplab variants. Lastly, our proposed method does not take as input any shape information, which maybe beneficial for brain tumor segmentation. Incorporating shape prior into our proposed method may further improve our brain tumor segmentation performance.

## Data Availability Statement

The original contributions presented in the study are publicly available. This data can be found here: https://github.com/sustecher/brain_tumor_segmentation/.

## Ethics Statement

The studies involving human participants were reviewed and approved by Research Ethics Committee of the First Affiliated Hospital of Sun Yat-sen University. The patients/participants provided their written informed consent to participate in this study.

## Author Contributions

This study was conceived and designed by YZ and XT. The experiments were performed by YZ, PZ, DJ, and JW. The clinical dataset was acquired by SZ and JC. Data were analyzed by YZ, YL, EW, and XT. The results were discussed by all authors. The manuscript was written by YZ and XT. All authors read and approved the final version of the manuscript.

## Funding

This work was supported by the National Natural Science Foundation of China (62071210), the Shenzhen Basic Research Program (JCYJ20190809120205578), the National Key R&D Program of China (2017YFC0112404), and the High-level University Fund (G02236002).

## Conflict of Interest

The authors declare that the research was conducted in the absence of any commercial or financial relationships that could be construed as a potential conflict of interest.

## Publisher's Note

All claims expressed in this article are solely those of the authors and do not necessarily represent those of their affiliated organizations, or those of the publisher, the editors and the reviewers. Any product that may be evaluated in this article, or claim that may be made by its manufacturer, is not guaranteed or endorsed by the publisher.

## Footnotes

^1^ https://hub.docker.com/r/sustechmedical/brain_tumor_segmentation/

^2^ https://github.com/sustecher/brain_tumor_segmentation/

^3^ https://ipp.cbica.upenn.edu/

## References

[1] Louis DN Perry A Reifenberger G Von Deimling A Figarella-Branger D Cavenee WK. The 2016 World Health Organization classification of tumors of the central nervous system: a summary. Acta Neuropathol. (2016) 131:803–20. 10.1007/s00401-016-1545-1 27157931

[2] Ostrom QT Gittleman H Xu J Kromer C Wolinsky Y Kruchko C. CBTRUS statistical report: primary brain and other central nervous system tumors diagnosed in the United States in 2009-2013. Neuro Oncol. (2016) 18:v1–75. 10.1093/neuonc/now207 28475809 PMC8483569

[3] Bakas S Akbari H Sotiras A Bilello M Rozycki M Kirby JS. Advancing the cancer genome atlas glioma MRI collections with expert segmentation labels and radiomic features. Sci Data. (2017) 4:170117. 10.1038/sdata.2017.117 28872634 PMC5685212

[4] Menze BH Jakab A Bauer S Kalpathy-Cramer J Farahani K Kirby J. The multimodal brain tumor image segmentation benchmark (BRATS). IEEE Trans Med Imaging. (2014) 34:1993–2024. 10.1109/TMI.2014.2377694 25494501 PMC4833122

[5] Bakas S Reyes M Jakab A Bauer S Rempfler M Crimi A. Identifying the best machine learning algorithms for brain tumor segmentation, progression assessment, and overall survival prediction in the BRATS challenge. arXiv preprint arXiv:181102629. (2018).

[6] Bakas S Akbari H Sotiras A Bilello M Rozycki M Kirby J. Segmentation labels and radiomic features for the pre-operative scans of the TCGA-LGG collection. Cancer Imaging Arch. (2017) 286. 10.1038/sdata.2017.117 PMC5685212 28872634

[7] Njeh I Sallemi L Ayed IB Chtourou K Lehericy S Galanaud D. 3D multimodal MRI brain glioma tumor and edema segmentation: a graph cut distribution matching approach. Comput Med Imaging Graph. (2015) 40:108–19. 10.1016/j.compmedimag.2014.10.009 25467804

[8] Wu J Zhang Y Tang X. Simultaneous tissue classification and lateral ventricle segmentation via a 2D U-net driven by a 3D fully convolutional neural network. In: 2019 41st Annual International Conference of the IEEE Engineering in Medicine and Biology Society (EMBC). Berlin (2019). p. 5928–31. 10.1109/EMBC.2019.8856668 31947198

[9] Kao PY Shailja F Jiang J Zhang A Khan A Chen JW. Improving patch-based convolutional neural networks for MRI brain tumor segmentation by leveraging location information. Front Neurosci. (2020) 13:1449. 10.3389/fnins.2019.01449 32038146 PMC6993565

[10] Dolz J Gopinath K Yuan J Lombaert H Desrosiers C Ayed IB. HyperDense-Net: a hyper-densely connected CNN for multi-modal image segmentation. IEEE Trans Med Imaging. (2018) 38:1116–26. 10.1109/TMI.2018.2878669 30387726

[11] Ghaffari M Sowmya A Oliver R. Automated brain tumor segmentation using multimodal brain scans: a survey based on models submitted to the BraTS 2012-2018 challenges. IEEE Rev Biomed Eng. (2019) 13:156–68. 10.1109/RBME.2019.2946868 31613783

[12] Ronneberger O Fischer P Brox T. U-net: Convolutional networks for biomedical image segmentation. In: Medical Image Computing and Computer-Assisted Intervention – MICCAI 2015. Cham (2015). 9351:234–41. 10.1007/978-3-319-24574-4_28

[13] Falk T Mai D Bensch R Çiçek Ö Abdulkadir A Marrakchi Y. U-Net: deep learning for cell counting, detection, and morphometry. Nat Methods. (2019) 16:67–70. 10.1038/s41592-018-0261-2 30559429

[14] Zhang Y Wu J Liu Y Chen Y Chen W Wu EX. A deep learning framework for pancreas segmentation with multi-atlas registration and 3D level-set. Med Image Anal. (2021) 68:101884. 10.1016/j.media.2020.101884 33246228

[15] Sinha A Dolz J. Multi-scale self-guided attention for medical image segmentation. IEEE J Biomed Health Inform. (2020) 25:121–30. 10.1109/JBHI.2020.2986926 32305947

[16] Shen H Wang R Zhang J McKenna SJ. Boundary-aware fully convolutional network for brain tumor segmentation. In: Medical Image Computing and Computer-Assisted Intervention? MICCAI 2017. Cham (2017). p. 433–41. 10.1007/978-3-319-66185-8_49

[17] Hu Y Liu X Wen X Niu C Xia Y. Brain tumor segmentation on multimodal MR imaging using multi-level upsampling in decoder. In: International MICCAI Brainlesion Workshop. Granada (2018). p. 168–77. 10.1007/978-3-030-11726-9_15

[18] Caver E Chang L Zong W Dai Z Wen N. Automatic brain tumor segmentation using a U-net neural network. In: International MICCAI Brainlesion Workshop. Granada (2018). p. 63–73.

[19] Choudhury AR Vanguri R Jambawalikar SR Kumar P. Segmentation of brain tumors using DeepLabv3+. In: International MICCAI Brainlesion Workshop. Granada (2018). p. 154–67. 10.1007/978-3-030-11726-9_14

[20] McKinley R Meier R Wiest R. Ensembles of densely-connected CNNs with label-uncertainty for brain tumor segmentation. In: International MICCAI Brainlesion Workshop. Granada (2018). p. 456–65. 10.1007/978-3-030-11726-9_40

[21] Razzak MI Imran M Xu G. Efficient brain tumor segmentation with multiscale two-pathway-group conventional neural networks. IEEE J Biomed Health Inform. (2018) 23:1911–9. 10.1109/JBHI.2018.2874033 30295634

[22] Sun L Zhang S Chen H Luo L. Brain tumor segmentation and survival prediction using multimodal MRI scans with deep learning. Front Neurosci. (2019) 13:810. 10.3389/fnins.2019.00810 31474816 PMC6707136

[23] Kao PY Ngo T Zhang A Chen JW Manjunath B. Brain tumor segmentation and tractographic feature extraction from structural MR images for overall survival prediction. In: International MICCAI Brainlesion Workshop. Granada (2018). p. 128–41. 10.1007/978-3-030-11726-9_12

[24] Feng X Tustison NJ Patel SH Meyer CH. Brain tumor segmentation using an ensemble of 3D U-Nets and overall survival prediction using radiomic features. Front Comput Neurosci. (2020) 14:25. 10.3389/fncom.2020.00025 32322196 PMC7158872

[25] Luo Z Jia Z Yuan Z Peng J. HDC-Net: hierarchical decoupled convolution network for brain tumor segmentation. IEEE J Biomed Health Inform. (2020). 10.1109/JBHI.2020.2998146 32750914

[26] Isensee F Kickingereder P Wick W Bendszus M Maier-Hein KH. No new-net. In: International MICCAI Brainlesion Workshop. Granada (2018). p. 234–44. 10.1007/978-3-030-11726-9_21

[27] Wang Y Peng J Jia Z. Brain tumor segmentation via C-dense convolutional neural network. Prog Artif Intell. (2021) 10:147–159. 10.1007/s13748-021-00232-8

[28] Myronenko A. 3D MRI brain tumor segmentation using autoencoder regularization. In: International MICCAI Brainlesion Workshop. Granada (2018). p. 311–20. 10.1007/978-3-030-11726-9_28

[29] Zhu J Li Y Hu Y Ma K Zhou SK Zheng Y. Rubik's Cube+: a self-supervised feature learning framework for 3D medical image analysis. Med Image Anal. (2020) 64:101746. 10.1016/j.media.2020.101746 32544840

[30] Ciompi F de Hoop B van Riel SJ Chung K Scholten ET Oudkerk M. Automatic classification of pulmonary peri-fissural nodules in computed tomography using an ensemble of 2D views and a convolutional neural network out-of-the-box. Med Image Anal. (2015) 26:195–202. 10.1016/j.media.2015.08.001 26458112

[31] Zhou Y Xie L Shen W Wang Y Fishman EK Yuille AL. A fixed-point model for pancreas segmentation in abdominal CT scans. In: Medical Image Computing and Computer Assisted Intervention? MICCAI 2017. Cham (2017). p. 693–701. 10.1007/978-3-319-66182-7_79

[32] Roth HR Lu L Seff A Cherry KM Hoffman J Wang S. A new 2.5 D representation for lymph node detection using random sets of deep convolutional neural network observations. In: Medical Image Computing and Computer-Assisted Intervention – MICCAI 2014. Cham (2014). p. 520–7. 10.1007/978-3-319-10404-1_65 PMC4295635 25333158

[33] Isensee F Jaeger PF Kohl SA Petersen J Maier-Hein KH. nnU-Net: a self-configuring method for deep learning-based biomedical image segmentation. Nat Methods. (2020). 10.1038/s41592-020-01008-z 33288961

[34] Nema S Dudhane A Murala S Naidu S. RescueNet: An unpaired GAN for brain tumor segmentation. Biomed Signal Process Control. (2020) 55:101641. 10.1016/j.bspc.2019.101641

[35] Wang W Chen C Ding M Li J Yu H Zha S. TransBTS: multimodal brain tumor segmentation using transformer. arXiv preprint arXiv:210304430. (2021). 10.1007/978-3-030-87193-2_11

[36] Aziz MJ Farnia P Alimohamadi M Makkiabadi B Ahmadian A Alirezaie J. Accurate automatic glioma segmentation in brain MRI images based on CapsNet. bioRxiv. (2021). 10.1101/2021.07.03.450931 34892080

[37] Dalca AV Sridharan R Cloonan L Fitzpatrick KM Kanakis A Furie KL. Segmentation of cerebrovascular pathologies in stroke patients with spatial and shape priors. Med Image Comput Comput Assist Interv. (2014) 17:773–80. 10.1007/978-3-319-10470-6_96 25485450 PMC4260817

[38] Wu J Tang X. Brain segmentation based on multi-atlas and diffeomorphism guided 3D fully convolutional network ensembles. Pattern Recogn. (2021) 115:107904. 10.1016/j.patcog.2021.107904

[39] Mahbod A Chowdhury M Smedby Ö Wang C. Automatic brain segmentation using artificial neural networks with shape context. Pattern Recogn Lett. (2018) 101:74–9. 10.1016/j.patrec.2017.11.016

[40] Brusini I Lindberg O Muehlboeck J Smedby Ö Westman E Wang C. Shape information improves the cross-cohort performance of deep learning-based segmentation of the hippocampus. Front Neurosci. (2020) 14:15. 10.3389/fnins.2020.00015 32226359 PMC7081773

[41] Zhang Y Wu J Liu Y Chen Y Wu E Tang X. MI-UNet: Multi-inputs UNet incorporating brain parcellation for stroke lesion segmentation from T1-weighted magnetic resonance images. IEEE J Biomed Health Inform. (2020) 25:526–35. 10.1109/JBHI.2020.2996783 32750908

[42] Li X Chen H Qi X Dou Q Fu CW Heng PA. H-DenseUNet: hybrid densely connected UNet for liver and tumor segmentation from CT volumes. IEEE Trans Med Imaging. (2018) 37:2663–74. 10.1109/TMI.2018.2845918 29994201

[43] Jia H Song Y Huang H Cai W Xia Y. HD-net: Hybrid discriminative network for prostate segmentation in MR images. In: Medical Image Computing and Computer Assisted Intervention – MICCAI 2019. Cham (2019). p. 110–8. 10.1007/978-3-030-32245-8_13

[44] Wu J Zhang Y Tang X. A joint 3D+2D fully convolutional framework for subcortical segmentation. In: Med. Image Comput. Comput. Assist. Interv. Shenzhen (2019). p. 301–9. 10.1007/978-3-030-32248-9_34

[45] Ren ZL Shen D Wang Q. Ensembles of multiple scales, losses and models for brain tumor segmentation and overall survival time prediction task. In: International MICCAI Brainlesion Workshop. Granada (2018). p. 402–10.

[46] Banerjee S Mitra S Shankar BU. Single seed delineation of brain tumor using multi-thresholding. Inf Syst. (2016) 330:88–103. 10.1016/j.ins.2015.10.018

[47] Jiang Z Ding C Liu M Tao D. Two-stage cascaded U-Net: 1st place solution to brats challenge 2019 segmentation task. In: International MICCAI Brainlesion Workshop. Shenzhen (2019). 11992:231–41. 10.1007/978-3-030-46640-4_22

[48] Isensee F Jaeger PF Full PM Vollmuth P Maier-Hein KH. nnU-Net for brain tumor segmentation. arXiv preprint arXiv:201100848. (2020). 10.1007/978-3-030-72087-2_11

[49] Jia H Cai W Huang H Xia Y. H2NF-Net for brain tumor segmentation using multimodal MR imaging: 2nd place solution to BraTS challenge 2020. Segmentation Task. (2020). 10.1007/978-3-030-72087-2_6

[50] Menze B Isensee F Wiest R Wiestler B Maier-Hein K Reyes M. Analyzing magnetic resonance imaging data from glioma patients using deep learning. Comput Med Imaging Graph. (2021) 88:101828. 10.1016/j.compmedimag.2020.101828 33571780 PMC8040671

[51] Woolrich MW Jbabdi S Patenaude B Chappell M Makni S Behrens T. Bayesian analysis of neuroimaging data in FSL. Neuroimage. (2009) 45:S173–86. 10.1016/j.neuroimage.2008.10.055 19059349

[52] Smith SM Jenkinson M Woolrich MW Beckmann CF Behrens TE Johansen-Berg H. Advances in functional and structural MR image analysis and implementation as FSL. Neuroimage. (2004) 23:S208–19. 10.1016/j.neuroimage.2004.07.051 15501092

[53] Jenkinson M Beckmann CF Behrens TE Woolrich MW Smith SM. FSL. Neuroimage. (2012) 62:782–90. 10.1016/j.neuroimage.2011.09.015 21979382

[54] Zhao J Wang Y Li X Hu M Li Z Song Y. Comparative analysis of the diffusion kurtosis imaging and diffusion tensor imaging in grading gliomas, predicting tumour cell proliferation and IDH-1 gene mutation status. J Neurooncol. (2019) 141:195–203. 10.1007/s11060-018-03025-7 30414095

[55] Young RM Jamshidi A Davis G Sherman JH. Current trends in the surgical management and treatment of adult glioblastoma. Ann Transl Med. (2015) 3. 26207249 10.3978/j.issn.2305-5839.2015.05.10 PMC4481356

